# A qualitative exploration of the pathway to diagnosis and treatment of cutaneous squamous cell carcinoma of the head and neck with perineural spread

**DOI:** 10.1002/cam4.70118

**Published:** 2024-08-23

**Authors:** Poorva Pradhan, Ke (Zoe) Wan, Li Chan, Tsu‐Hui (Hubert) Low, Raymond Wu, Jenny H. Lee, Carsten E. Palme, Jonathan R. Clark, Rebecca L. Venchiarutti

**Affiliations:** ^1^ Department of Head and Neck Surgery Chris O'Brien Lifehouse Camperdown New South Wales Australia; ^2^ Sydney School of Public Health, The University of Sydney Sydney New South Wales Australia; ^3^ Department of Radiation Oncology St George Hospital Kogarah New South Wales Australia; ^4^ Central Clinical School, Faculty of Medicine and Health, University of Sydney Sydney New South Wales Australia; ^5^ Department of Otolaryngology–Head & Neck Surgery Faculty of Medicine and Health Sciences, Macquarie University Sydney New South Wales Australia; ^6^ Department of Radiation Oncology Chris O'Brien Lifehouse Camperdown New South Wales Australia; ^7^ Department of Medical Oncology Chris O'Brien Lifehouse Camperdown New South Wales Australia; ^8^ Faculty of Medicine and Health Sciences Faculty of Medicine and Health Sciences, Macquarie University Sydney New South Wales Australia; ^9^ Royal Prince Alfred Institute of Academic Surgery, Sydney Local Health District Camperdown New South Wales Australia

**Keywords:** cutaneous squamous cell carcinoma, head and neck, models of pathways to treatment, neoplasm, Perineural spread

## Abstract

**Background:**

Perineural spread (PNS) is associated with a poor prognosis in cutaneous squamous cell carcinoma of the head and neck (cSCCHN). Hence, investigating facilitators and barriers of early diagnosis and treatment of PNS in cSCCHN may improve outcomes.

**Methods:**

Patients were recruited from an institutional database. Semi‐structured interviews were conducted according to the Model of Pathways to Treatment. Thematic analysis was based on the four main intervals in the framework using a data‐driven analytical method.

**Results:**

Seventeen participants were interviewed. Facilitators included patients' past experiences, symptom progression, trust in healthcare professionals (HCPs), and capacity to leverage relationships. Barriers included difficult diagnoses, limited access to cancer services, lack of care coordination, and lack of awareness of PNS among primary health care providers.

**Conclusion:**

These findings emphasise the complexity early diagnosis and treatment of PNS. Interventions like clinical practice guidelines, education for HCPs, and telehealth could facilitate timely detection and management.

## 1 INTRODUCTION

Cutaneous squamous cell carcinoma (cSCC) is the second most common non‐melanoma skin cancer worldwide, following basal cell carcinoma (BCC).^1^, ^2^, ^3^ The Australian Institute of Health and Welfare estimates that nearly two‐thirds of Australians may be diagnosed with a non‐melanoma skin cancer before 70 years of age.^4^ The primary treatment for cSCC is surgical excision, with alternative treatments including radiotherapy, immunotherapy,^1^ ablative therapies such as cryotherapy, and topical 5‐fluorouracil.^5^ In general, the prognosis of cSCC is excellent, with five‐year survival rates exceeding 90%.^1^, ^6^, ^7^

Perineural spread (PNS) refers to the retrograde (or centripetal) spread of a malignancy away from the primary tumour within the peri‐ and endoneural spaces of large named nerves.^8^ The diagnosis of PNS is based on clinical and radiological features and differs from perineural invasion (PNI), which is a pathological diagnosis. In the head and neck, the branches of the facial and trigeminal nerves are most often affected.^9^, ^10^ The extent of anatomical spread is based on a ‘Zonal Classification’ system which is defined by the proximity of disease to the brainstem with ‘Zone 3’ disease having the worst prognosis.^11^, ^12^ Therefore, early diagnosis and identification of PNS is critical to improve treatment response, reduce recurrence, and increase survival.^13^

PNS in the head and neck usually presents as either a slowly progressive dysaesthesia affecting one or more branches of the trigeminal nerve or a slowly progressive facial nerve palsy. For the latter, it is important to distinguish from Bell's palsy, which has a sudden onset.^8^ However, PNS is often misdiagnosed, leading to delays in diagnosis and treatment.^10^, ^14^ Several Australian studies have reported the timeliness of diagnosis and treatment of PNS from cSCC of the head and neck (cSCCHN). The median time from symptom onset to diagnosis of PNS in these case series has been reported as 6 months (range 2 weeks–5 years),^15^ 8.9 months (range 0.5–48 months),^14^ and 12 months (range 3–62 months).^16^ However, these studies do not provide detailed insight into the events occurring over this time period of delayed diagnosis, which includes decisions made by both people with PNS as well as healthcare professionals involved in assessment and diagnosis.

Several theoretical models have been proposed to describe the events that underpin the pathway to cancer diagnosis.^17^ For instance, the *Model of Pathways to Treatment*, conceptualised by Walter and colleagues is one such framework that delineates the route to cancer diagnosis and treatment.^18^ This model primarily focuses on patient factors in the appraisal of symptoms and subsequent help seeking for these symptoms (see Scott et al 2013 for a detailed account).^19^ This framework is useful to explore factors that contribute to events along the diagnostic pathway. Therefore, the aim of the current study to conduct an in‐depth exploration of the events along the pathway to treatment for people with cSCCHN with PNS, to identify potential aspects of the pathway that can be optimised to improve patient outcomes.

## 2 METHODS

### 2.1 Study design and setting

The study adopted a qualitative design consisting of semi‐structured interviews with participants who had a history of cSCCHN with PNS. The interview guide was structured in accordance with the *Model of Pathways to Treatment*,^18^ a framework known for its efficacy in comprehensively investigating events along the pathway to diagnosis of symptomatic cancer.^19^

Chris O'Brien Lifehouse is a comprehensive cancer centre in New South Wales, Australia. NSW is the most populous state/territory in Australia, with approximately 8.15 million residents.^20^ Australia has both private and public (universal) health care systems, allowing patients to access primary healthcare practitioners (HCPs) for cancer investigations and referrals to specialists.^21^ The Chris O'Brien Lifehouse hosts the Sydney Head and Neck Cancer Institute (SHNCI) database, containing information on more than 14,000 patients with head and neck cancer.

### 2.2 Participants

Participants were identified from the SHNCI database. Individuals were eligible to participate if they were aged ≥18 years, had a diagnosis of cSCCHN with clinical and/or radiologic evidence of PNS, and were treated between January 2010 and August 2021 inclusive. The exclusion criterion in this study was cognitive impairments that precluded participation in the interviews. Eligible patients were invited to participate through a mailed letter of invitation from their attending medical officers. Interested participants provided their written consent by reply‐paid post.

### 2.3 Consent

All participants completed the consent form and returned it to the study coordinators. Audio recordings of all interviews were made with consent from all participants.

### 2.4 Ethical approval

This study was approved by the Sydney Local Health District Human Research Ethics Committee (RPA Zone) (Protocol No. X23‐0069 & 2023/ETH00364) and site governance was approved by the Research Governance Office at Chris O'Brien Lifehouse (LH23.022).

### 2.5 Data collection

Semi‐structured interviews were conducted with participants in‐person, via video conferencing (Zoom, Version 5.16.10, California, United States), or by telephone to ensure the inclusion of participants residing in rural areas, as well as those who were outside routine follow‐up. The interviews were conducted by researchers (PP and RV) between August and September 2023. While most interviews were conducted individually, in one case, a participant's partner was present and contributed to the interview.

Before conducting interviews, interviewers were thoroughly informed about the *Model of Pathways to Treatment* via comprehensive literature review. All participants were interviewed once; no interviews were repeated. Participants were asked to recall their past experiences, starting from the initial signs or symptom to their treatment for cSCCHN with PNS, following a structured set of interview probes aligned with the four primary intervals of the *Model of Pathways to Treatment*. These intervals included symptom appraisal, help‐seeking, diagnosis, and pre‐treatment.^17^ Facilitators and barriers for early diagnosis and treatment were evaluated based on participants' narratives of their medical journey.

At the end of the interview, the audio recordings were reviewed and transcribed, and participants were given the opportunity to review their transcript. Interviews were undertaken until data saturation (no new themes emerged from interviews).^22^ The electronic medical records of participants were reviewed to collect clinical data, such as events along the pathway to treatment which were used to verify or triangulate the information from the interview. Clinical data was obtained from electronic medical records and the SHNCI database.

### 2.6 Data analysis

The Transcription Software ‘Otter AI’ (https://otterai/home; California, United States) was used to transcribe all interview recordings. One author (PP) checked all transcripts to ensure accuracy. The transcriptions were analysed using NVivo software (version 14.23.2, Lumivero, Denver, United States) and data were analysed thematically. Meaning units in the original texts were identified from transcriptions, and these were generalised into compression units that maintained the meaning of the original text. These compression units were assigned specific codes, with the authors comparing these codes to group them into different themes. An initial coding framework was developed for the dataset after discussions among three authors (PP, ZW and RV) based on the first three interviews. Differences in the development of codes were resolved and discussed at regular meetings. The remaining transcripts were coded using this framework, which was revised as data were analysed by two researchers (PP and ZW). A third researcher (RV) did the final check for each interview transcript.

## 3 RESULTS

### 3.1 Participant characteristics

A total of 64 patients were identified as eligible and were invited to participate. Of these, 22 returned the consent form expressing their interest in participating and 17 participants (mean age 70 years; *n* = 13 males) were interviewed. The remaining five who provided consent were not interviewed due to data saturation. No new themes or patterns emerged in the fifteenth interview, therefore two additional interviews were conducted to confirm data saturation. Interviews lasted between 45 and 60 min (mean duration 53 min). The majority of participants had PNS involving the trigeminal nerve only (65%; *n* = 11), two participants had involvement of the facial nerve only (12%), and the remaining four participants (23%) had involvement of both the trigeminal and facial nerves. Table 1 summarises the characteristics of participants.

**TABLE 1:** Participant demographics.

Characteristic	*N* (%)
Mean age	70 years
Gender	
Male	13 (76%)
Female	4 (24%)
Geographical location	
Major cities	8 (47%)
Inner regional	8 (47%)
Outer regional	1 (6%)
Type of nerve involved	
Trigeminal only	11 (65%)
Facial only	2 (12%)
Both (trigeminal & facial)	4 (23%)
Time since diagnosis (PNS recurrence)	
<1 year	3 (18%)
1–2 years	9 (53%)
2–3 years	5 (29%)
Treatment history	
Immunotherapy	15 (88%)
Surgery	15 (88%)
Radiation	13 (77%)
Chemotherapy	1 (6%)
Treatment intent	
Curative	11 (65%)
Palliative	6 (35%)

### 3.2 Themes

The findings revealed four major themes: awareness, appraisal, barriers, and facilitators (Table 2). Within each theme are sub‐themes that illustrate factors from participants' perspectives that influenced their pathway to diagnosis and treatment. Table 2 provides exemplar quotations for each of the sub‐themes.

**TABLE 2:** Key themes identified describing facilitators and barriers to early diagnosis and treatment of PNS in cSCC patients with supporting quotations.

Theme *Subtheme*	Supporting quotations [*Gender, Age, Nerve Involved*]
Awareness	
*A. Awareness of PNS among primary healthcare providers*	‘So I talked to my GP. He wasn't too concerned because again, he couldn't see anything.’ [*Male, 62 years, CNV2* (*infraorbital nerve*)] ‘No, he didn't know anything about it. Though, he was just more concerned about the Bell's Palsy. You know, because I went to him for the Bell's Palsy. And he gave me some medication and it didn't help.’ [*Female, 84 years, CNV3*]
*B. Specialised knowledge*	‘The Professor said that was a tumour. But he said it wasn't Bell's Palsy at all…he said yes. It's on V1 and V2 of the [trigeminal] nerve.’ [*Male, 55 years, CNV2* (*infraorbital nerve*)] ‘So I had to go down to her…Yeah. So went down there. She read the MRI and then I came home and then she rang me back and said we need to do a nerve operation.’ [*Female, 64 years, CNV2 (infraorbital nerve*)]
*C. Educating patients about PNS*	‘He told me about the operation they'd be doing…He said that [the cancer] would come back. He said it was common for all those things to happen with it, that they would come back in a couple of months…Yes, he was easy to understand. He explained it very well.’ [*Male, 78 years, CNV3*]
*D. History of skin cancer and undergoing regular skin checks*	‘I've had a few BCCs…So I see a dermatologist every couple of months as well for my check‐up…six month[ly] appointments with my skin cancer doctor…’ [*Male, 65 years, CNVII*] ‘We used to have a 12 monthly check‐up on that… If not, sometimes sooner if I had something suspicious [detected] by my GP who was qualified to do a skin check.’ [*Male, 72 years, CNVII*]
Appraisal	
*A. Initial signs and symptoms*	‘Pins and needles and numbness on my cheek and chin.’ [*Male, 72 years, CNVII*] ‘It started with [an] electric shock… just like ants and things crawling under his skin.’ [*Male, 55 years, CNV2* (*infraorbital nerve*)] ‘I could feel like a little lump which turned out to be this thickening from what I understand.’ [*Male, 65 years, CNV1* (*supraorbital and supratrochlear nerves*)] ‘[A] very hard lump…nearly right under the eye.’ [*Male, 65 years, CNVII*] ‘I pointed out a bit of a spot below my right eye… [like a] worm crawling…’ [*Male, 67 years, CNV2* (*infraorbital nerve*)] ‘Just a numb feeling in the left side of the jaw, lower jaw…no it's all internal all everything was internal.’ [*Male, 77 years, CNV3*]
*B. Rapid progression or development of new symptoms*	‘I [was] still get getting headaches and my eye was getting worse and my right side of my face was still numb all the way down…it was growing…my face was all numb.’ [*Male, 65 years, CNVII*] ‘[I] started getting shooting pain up to my right eye and running up to the side of my upper nose…some more pain and coming back every six months.’ [*Male, 67 years, CNV2* (*infraorbital nerve*)]
*C. Gut feeling*	‘I just had a gut feeling that something wasn't right.’ [*Male, 55 years, CNV2* (*infraorbital nerve*)] ‘Because I kept saying there's something there. There's something there and I couldn't figure out what it was.’ [*Male, 77 years, CNV3*]
*D. Non‐patient appraisal of signs and symptoms*	‘My wife came to me and said yeah, this is not right…She was very concerned that something happening. So yeah. We have to get to the bottom of it.’ [*Male, 65 years, CNVII*]
Barriers	
*A. Difficult diagnosis and ‘unusual cases’*	‘And he said, you just got stressed and it will go away…The neurologist said that it was Bell's Palsy and to come home and do a diary…And he said there was nothing there it was virtually all in my head and in the headaches.’ [*Male, 55 years, CNV2* (*infraorbital nerve*)] ‘I kept saying to the GP and GP told [me] to go to the dentist, I went to the dentist. He took X rays. There's nothing there. He sent me to an oral surgeon. He did all the tests and X rays. He said there's nothing there. [*Male, 77 years, CNV3*]
*B. Misdiagnosis*	‘And he sent us to the neurologist thinking that it was the Bell's Palsy, migraines. And then, of course, the neurologist sent us away.’ [*Male, 55 years, CNV2* (*infraorbital nerve*)] ‘[The] neurologist … thought it was my trigeminal nerve…Trigeminal nerve compression, and there is some doubt that might have been the light might have been a misdiagnosis.’ [*Male, 72 years, CNVII*]
*C. Access to healthcare services and specialists*	‘It's very difficult to go to the doctor around here…but you've sort of got to wait two weeks to get into a doctor…We're 500 to 600 kilometres and takes six hours to drive straight with without a stop…there was a two week waiting list, getting to see my GP…And to see a specialist…takes months, months and months.’ [*Female, 68 years, CNV2* (*infraorbital nerve*)] ‘Yes, it is a big gap. And it was all because of the COVID…I had to make an appointment…[if] COVID hadn't been there, I would probably have been able to get my lower lip seen to quicker. Okay. But because of the COVID and misreading the whole thing. It's sort of delayed things. So, I think if COVID if there was no COVID I would have probably got on to this quicker.’ [*Female, 84 years, CNV3*]
*D. Lack of care coordination*	‘I'm not sure how long my dermatologist, just the previous one was actually reporting back to him. Because I had quite a lot done, but I'm not sure he was certainly hearing…I don't think he's necessarily hearing back very well, from that I don't have all those records, to decent dermatologists.’ [*Male, 62 years, CNV2* (*infraorbital nerve*)]
Facilitators	
*A. Trust of HCPs*	‘Um, we've got a very, very good GP he comes to the house if we need to get a great relationship with him…just the fact that we trusted his advice and went with it.’ [*Male, 55 years, CNV2* (*infraorbital nerve*)] ‘Yeah, they're really nice [and] accommodating and just keep me informed of everything that's going on and let me know everything. They're really good. Yeah, sure…Because of the professionalism of all of the doctors and specialists that I've seen.’ [*Male, 71 years, CNV1* (*supraorbital nerve*)]
*B. Self‐management of health and self‐advocacy*	‘So we made an appointment to see my skin specialist as soon as we got home…I kept going back and nagging him to do something.’ [*PNS 63, M, 65 years, CNVII*] ‘And just when I went to my check‐up, I just said to [partner], there was something there and we had the scan…I want to get it done quick. Get it out. So I knew you had to operate fast. Yes. So I'd had cancer while I was still being treated for cancer. I know time is precious. You've got to act as quick as you can.’ [*Female, 68 years, CNV2* (*infraorbital nerve*)]
*C. Sense of urgency*	‘My dermatologist was very aware. So she had mechanism started and she would have referred me into [Hospital] most likely anyway, or possibly other places…so you being an unusual sort of cancer, then it's like, okay, well, this really is life threatening if it continues.’ [*Male, 62 years, CNV2* (*infraorbital nerve*)] ‘And then he got me into the operation not long after that as soon as possible. When I was diagnosed with being malignant, he got me into [Hospital] asked me straight away…I get some scans done or something straightaway.’ [*Male, 78 years, CNV3*]
*D. Referral processes*	‘Then my GP finally sent me to a neurologist…And go to the dentist, go to the GP went to the oral surgeon.*’* [*Male, 77 years, CNV3*] ‘And he said there was something at the back there, he couldn't identify it…And I then went to a [Surgeon 1] down at [Hospital]…he suggested to go and see a neurosurgeon…And I finally went back to Sydney and [Surgeon 2] referred me to another specialist.’ [*Male, 82 years, CNV2* (*infraorbital nerve*)]
*E. Access to cancer services*	‘We're going to operate on it wait one week … It's a couple of hours out of your day. That's about it and it was not really far.’ [*Male, 65 years, CNVII*] ‘Especially with [Surgeon] it was very quick… [Hospital] kept a diary of [Patient]'s symptoms and our timeline of what's happened and got as much information as I could the MRI result. And within two weeks we were down in Sydney…we don't travel to Sydney for treatment…Then he was operated on two weeks after that … And then two weeks after he started immunotherapy two weeks.’ [*Male, 55 years, CNV2* (*infraorbital nerve*)]
*F. Leveraging relationships or social networks*	‘But I was actually very fortunate to have worked for [Company], a colleague of mine, and her husband was a head and neck cancer surgeon…He referred me into [Surgeon] straight up. Right. It was really quick.’ [*Male, 62 years, CNV2* (*infraorbital nerve*)] ‘He referred me to a friend of his…who was a…surgeon. And he was a nice guy, and he might have as a dentist, so to come across that sort of thing because they know all about the nerves in the face as well affecting teeth and send me to an ophthalmic friend…’ [*Male, 74 years, CNVII*]

### 3.3 Theme 1: Awareness

#### 3.3.1 Awareness of PNS among primary healthcare providers

Usually, general practitioners (GPs) were the first HCP that participants saw for assistance with symptoms. Given the relative rarity of PNS and the non‐specific symptoms experienced by participants, many participants noted that their GP did not initially suspect PNS. The misattribution of signs or symptoms of PNS to other conditions such as Bell's palsy (when facial droop was present) or trigeminal neuralgia (when dysaesthesia was present) led to prolonged diagnostic intervals for many participants. Given the absence of a primary lesion in some cases, participants felt that this hindered timely and accurate diagnosis, because their symptoms were “invisible”. For example, one participant reported facial numbness to his GP, but because there were no other clinical signs, their GP did not express concern or investigate further at that stage. Additionally, some participants suggested that cultural factors played a role. Participants felt that some South Asian HCPs had limited knowledge about skin cancers and were even less aware about PNS, possibly because skin cancers are uncommon in people with darker skin.^23^

#### 3.3.2 Specialised knowledge

Accurate and timely detection of symptoms depended on the expertise of HCPs. One participant reported numbness to their GP, who specialised in skin cancer, leading to a prompt diagnosis of PNS. Participants whose GPs had established a correct diagnosis themselves felt their referral to specialist head and neck surgeons was expedited and subsequently PNS was swiftly managed. However, paradoxically, sometimes a misdiagnosis occurred based on the HCPs specialist knowledge, which could then prolong the diagnostic interval. One participant, who presented with a dysesthesia (described as the sensation of “insects crawling” on their skin), was diagnosed with Bell's palsy by both their GP and neurologist. However, several months later after review of the initial MRI, a head and neck surgeon made a diagnosis of PNS. Thus, specific knowledge about PNS was important for HCPs to facilitate early diagnosis, and HCPs should be alert to signs and symptoms of PNS such as facial droop, numbness, and shooting pain or other sensations which could be mistaken for Bell's palsy or trigeminal neuralgia.^16^

#### 3.3.3 Educating patients about PNS

Informing patients about PNS in the context of cSCC was a crucial aspect for enhancing patient awareness and facilitating diagnosis. One participant stated that his neurologist clearly informed him that PNS could be the cause for the symptoms he was experiencing. Effectively conveying this information required specific communication techniques to ensure patient understanding. Participants noted that HCPs who used layman's terms instead of medical jargon to describe PNS helped them to better understand the disease process. This approach to communication was believed to promote positive engagement and foster good relationships with the medical team.

#### 3.3.4 History of skin cancer and undergoing regular skin checks

Fourteen participants raised their prior experiences with skin cancer, including history of BCCs, SCCs, melanoma, and Bowen's disease, in the context of their diagnosis with PNS. Some considered their experiences may have helped early diagnosis of PNS, as most patients were engaging in regular skin checks, which provided an opportunity to have signs and symptoms assessed. In the context of a consultation for skin cancer surveillance and screening, participants exhibiting concerning signs or symptoms found that investigation and referral were more likely to be expedited by their HCPs.

### 3.4 Theme 2: Appraisal

#### 3.4.1 Initial signs and symptoms

Signs and symptoms of PNS were broadly described by participants as either “visible” or “invisible”. Often, participants described the first symptom they experienced as being “weird” or one that they had never experienced before, such as numbness, shooting pain, and the “sensation of insects crawling on [the] face”. These symptoms were usually present in the absence of any signs or cutaneous lesions. Participants' responses to the symptoms they experienced varied greatly. Some quickly appraised their symptoms as requiring attention, such as in the case of one participant who detected a small lump under his right eye, for which he promptly consulted his skin cancer specialist. In contrast, some participants appeared reluctant to seek help. This was exemplified by one participant who described the “sensation of an insect crawling on his face” but did not decide to seek help for about 6 months. In some cases, the initial symptom was more non‐specific, such as one participant who experienced headaches and who reported not being concerned because he thought his headache was secondary to post‐traumatic stress disorder. Thus, some participants tended to associate symptoms with other causes, leading to a decision to put off seeking help for their symptoms. These differences could be attributed to variations in individuals' health concerns and self‐assessment, as the initial diagnosis predominantly relied on patients' self‐detection.^24^

#### 3.4.2 Rapid progression or development of new symptoms

Rapid changes in the symptom or the development of new symptoms on top of existing ones was often the catalyst for more urgent help‐seeking by the participant and investigations by HCPs.^25^ Examples of rapidly progressing symptoms included pain and numbness. For instance, in one participant, numbness initially manifested in a 1 cm area which rapidly extended to 2 cm. Likewise, another participant initially experienced pain starting in the eye which rapidly progressed down to the cheek and neck, prompting them to urgently seek medical care. Another participant reported that initally he experienced a “sensation of insects crawling on the face”, which six months later was followed by a sudden facial droop. In the majority of cases, noteworthy new symptoms like facial droop caused a sense of alarm and motivated them to seek urgent medical attention.

#### 3.4.3 Gut feeling

Patients relied on their intuition about their own health and their ability to identify and contextualise bodily changes that provided valuable clinical insights for HCPs.^26^ Many participants explicitly expressed having a “gut feeling that something was wrong”, which they acted on when they sought help. These participants tended to retain this feeling and self‐advocated for further investigation or referral before their diagnosis. For instance, a participant with facial numbness relied on their intuition to self‐advocate for further investigation. Initially consulting a GP, they pursued specialist referral to a dermatologist and neurologist. The neurologist promptly recognised the severity, referring the individual to a head and neck surgeon, resulting in a diagnosis of PNS. In one case, a participant's spouse validated these gut feelings based on their own knowledge and experiences. This participant's wife, who was a nurse with 24 years of experience, questioned the initial diagnosis of Bell's palsy, as she was aware that the typical recovery times of Bell's palsy differed from what her spouse was experiencing. Both participants and their caregivers utilised their intuition to appraise any bodily changes and at times this led to further action.

#### 3.4.4 Non‐patient appraisal of signs and symptoms

In nearly every instance, individuals with PNS were the initial observers of bodily changes; however, the involvement of family and friends in symptom recognition and encouragement to seek help was notable. Some participants deferred seeking assistance until prompted or supported by their family members or friends. In one case, a participant's spouse, alarmed by the presence of a facial droop, took the initiative to contact a specialist skin cancer clinic and schedule an appointment for her partner. Another participant described how their wife played an important role in communicating his health concerns to their HCP, which facilitated information gathering by the HCP and led to the ultimate diagnosis of PNS. This demonstrates the importance of caregivers or support persons for individuals who are not as proactive with their health and may require additional support to engage with the health system. The participant reflected that his wife also played an important role in ‘translating’ medical terminology into layman's terms aid communication with the HCP.

### 3.5 Theme 3: Barriers

#### 3.5.1 Difficult diagnoses and ‘unusual’ cases

Participants reported that the rarity of PNS posed a considerable challenge to timely diagnosis and that PNS was rarely suspected by GPs. Despite imaging and review by multiple specialists, participants often felt that their “invisible symptoms” were dismissed because there were no objective signs or lesions. In some cases, the results of multiple imaging scans were inconclusive, delaying the diagnosis of PNS. Participants stated they were often told their case was ‘unusual’, potentially as a way to explain to the participant why they had not yet received a diagnosis. It was likely that some HCPs had not seen a case of PNS or that their knowledge of PNS was limited.

#### 3.5.2 Misdiagnosis

Misdiagnosis was common for many participants, often as Bell's palsy (when the facial nerve was involved) or trigeminal neuralgia (when the trigeminal nerve was involved). These misdiagnoses came from both GPs and specialists (neurologists), despite the clinical presentations of Bell's palsy and trigeminal neuralgia being distinct from PNS.^8^ Of the participants who were aware that a misdiagnosis occurred acknowledged the diagnostic challenge that PNS often presents. In some cases, participants were critical, attributing current sequelae of the disease (in some cases incurability) or treatment (extensive resections or radiation therapy) to the delay that these misdiagnoses imparted.

#### 3.5.3 Access to healthcare services and specialists

Several participants lived in regional areas and described challenges in accessing services and specialists due to limited availability and long travel distances. One participant expressed the difficulties they experienced in securing appointments with his GP, while another participant had to travel long distances for an ultrasound‐guided needle biopsy, enduring prolonged waiting times for the arranged investigations. Most participants reported that long distances to receive healthcare services was a barrier for them, particularly for one who resided 45 km away from their local GP, and 150 km away from a neurologist, which was especially challenging when difficult diagnoses required multiple appointments and investigations. Another participant pointed out that these issues deter patients residing in regional and remote areas from seeking timely medical assistance, thereby impeding the diagnostic process. The challenges were exacerbated during the COVID‐19 pandemic, with physical distancing strategies such as lockdown measures negatively impacting patients' access to healthcare services.

#### 3.5.4 Lack of care coordination

Well‐coordinated care, which encompasses elements of both navigation and communication,^27^ is critical to ensure that people receive high‐quality and timely healthcare. Several participants expressed their concerns about the gaps in communication they observed between HCPs involved in managing their care, which led to misunderstandings and delays. One participant suspected his dermatologist might not have adequately shared relevant medical information, such as his skin cancer history, with other HCPs, which he thought may have led to a diagnostic delay. Participants also stated that at times, HCPs were unable to gather the full medical history of patients due to the overwhelming number of cases they handled. As one participant reported, she needed to repeat her medical history for multiple HCPs, indicating a breakdown in information sharing. This suggests a potential misunderstanding about the standard practice, where each clinician typically conducts a comprehensive medical history assessment rather than relying solely on notes from other healthcare providers.

### 3.6 Theme 4: Facilitators

#### 3.6.1 Trust of HCPs

Establishing trust with HCPs was crucial in participant's healthcare journeys, significantly influencing the act of help‐seeking and establishing a clear diagnosis. Many participants indicated they had developed strong, trusting relationships with their GPs or specialists. This facilitated positive communication between patients and HCPs, empowering patients to take more decisive action and seek timely care, thereby reducing the diagnostic interval. One participant expressed deep trust in her treating team, which was reflected in how they coordinated appointments and care for her, which facilitated timely treatment.

#### 3.6.2 Self‐management of health and self‐advocacy

Participants who reported being able to self‐manage their health also often reported a smoother diagnostic process.^28^ These participants often advocated for themselves during appointments, questioning diagnoses and seeking second opinions either from HCPs or advice from their friends and family members. This was exemplified by one participant, who booked an appointment with a skin specialist after noticing a small skin lesion and sought additional assistance from other HCPs when he experienced persistent numbness after it was removed. His proactive stance could be attributed to his history of skin cancers, which made him more actively involved in management of his health. In addition, the unusual nature of PNS symptoms also appeared to play a role in how participants managed their own health. One participant, who noticed an unusual “tingling sensation” near the mouth, took a proactive approach by consulting a HCP. This decision was influenced by her regular annual skin checks and previous history of SCC, ultimately leading to a diagnosis of PNS. Overall, patients' active engagement and advocacy for their health were important in contributing to the early diagnosis of cancer.

#### 3.6.3 Sense of urgency

The sense of urgency conveyed by HCPs played a crucial role in facilitating early cancer diagnosis and subsequent treatment. This urgency often stemmed from being what participants described as an “unusual case”, which they felt prompted the HCP to quickly initiate mechanisms to accelerate the diagnostic process. One participant reported that this sense of urgency came from her dermatologist, who identified something distinctly unusual (“a blood spot”) upon examination, which prompted an urgent biopsy. In some instances, patients' gut feelings were triggered by their own experiences, such as consistent numbness on their face, leading to a sense of urgency and conveyed this to GP. The participant reported that this triggered the GP to refer the participant for an urgent CT scan.

#### 3.6.4 Referral processes

The referral actions that an HCP undertook were critical in facilitating timely diagnosis of PNS. In all cases, referral from a GP to a secondary HCP such as a neurologist, ear, nose, and throat (ENT) surgeon or head and neck surgeon was needed to reach the final diagnosis. In some cases, a single referral to a specialist was sufficient, and thus a diagnosis could be made within days or weeks. However, many participants described having to consult several HCPs before receiving the final diagnosis of PNS. For example, one participant with facial numbness initially saw his GP, who referred them to a dentist, who arranged an X‐ray. After this X‐ray did not reveal the cause of the numbness, the participant was referred to an oral surgeon and then ultimately a neurologist, who made the diagnosis of PNS. This experience is in line with previous series published by our service showing all patients consulted a minimum of three HCPs prior to being seen by a head and neck surgeon,^16^ reflecting the challenges in identifying HCPs with the requisite knowledge of PNS to make a diagnosis.

#### 3.6.5 Access to cancer services

Access to cancer services, which encompasses availability of HCPs and healthcare services, distance to services, and timely availability of appointments was shown to vary significantly among this cohort. People living in rural areas are known to have additional challenges accessing healthcare services, which was reflected in the interviews. In contrast, participants living in major cities, having greater availability of HCPs and shorter travel times to services generally reported a more simplified process in accessing the appropriate healthcare services than those living in rural areas. However, the disparity mainly affected the diagnostic process, rather than the treatment process. Participants noted that having been diagnosed with PNS, commencement of treatment was rapid (coupled with a sense of urgency given the nature of PNS) regardless of geographic location. The co‐location of oncological services in one location, or the network of clinicians managing this disease may have facilitated rapid access to treatment among this cohort.

#### 3.6.6 Leveraging relationships or social networks

During the help‐seeking process, before a diagnosis has been established, participants frequently shared the signs and symptoms with families and friends, which elicited suggested action to take (from the family member or friend), or these individuals acted on behalf of a participant. This process, which has been referred to as ‘leveraging social capital’, can play an important part in timely access to healthcare, diagnosis, and treatment.^21^ One participant described the symptoms he was experiencing to a work colleague; this colleague then mentioned his experience to their partner, who happened to be a head and neck surgeon and expressed concern. This prompted the participant to undergo an urgent MRI, thus leading to a diagnosis of PNS. Another participant described their symptoms to a personal friend, who was a dentist with expertise in facial nerves, who facilitated a rapid referral to an ophthalmic surgeon, who made the ultimate diagnosis. Utilising social networks in some cases therefore led to interventions by others that facilitated timely diagnosis of PNS.

## 4 DISCUSSION

This study evaluated facilitators and barriers impacting the diagnosis and treatment of patients with PNS from cSCCHN in NSW, Australia. Patient‐reported facilitators included prior experiences with skin cancers or routine skin checks, the recognition of ‘alarming symptoms’, intuition or ‘gut feelings’, trust in HCPs, self‐advocacy, a sense of urgency among HCPs, availability of cancer services, specialists' professional knowledge, quick referral mechanisms, and the capacity to leverage personal connections. Within these facilitators, symptom appraisal by patients played a crucial role, influencing their decision‐making and consequently affecting the speed at which diagnosis and treatment were sought. The barriers identified by patients included inconclusive findings on imaging delaying diagnosis, limited access to cancer services for rural patients, lack of care coordination, and lack of awareness by HCPs regarding PNS leading to misdiagnosis. Moreover, the challenges associated with long‐distance travel, compounded by COVID‐19 restrictions were barriers for rural patients.

Symptom appraisal serves as a key factor in patients' help‐seeking behaviour. This aligns with the concept of the Model of Pathways to Treatment and the Common‐Sense Model of Illness Self‐Regulation, which posits that patients' recognition of bodily changes prompts them to take actions to address a potential health threat.^19^, ^21^ Meanwhile, more alarming symptoms, such as progression of numbness, lead to proactiveness and urgently seeking help.^28^, ^29^ These actions resulted from their self‐awareness, often based on past experience with skin cancers, engagement in regular skin checks and acting on their gut feeling. Research consistently highlights that HCPs extensive knowledge and clinical expertise in understanding patient's condition, play a crucial role in facilitating early diagnosis,^21^, ^30^, ^31^ but there is a scarcity of research concentrating on patients' gut feelings.^32^ The findings in this study show the importance of patients' and caregivers' gut feeling in promoting early diagnosis through prompt help‐seeking, consistent with previous studies.^21^, ^32^

In contrast to patients' proactive help‐seeking, symptom appraisal by HCPs was often cited by participants as a barrier that caused diagnostic delays. This was manifested in instances where HCPs did not promptly recognise or assess symptoms. Participants frequently described the process of confirming a diagnosis as challenging leading to misdiagnosis, owing to the rarity PNS. For instance, HCPs often misdiagnosed PNS as Bell's Palsy or trigeminal neuralgia due to the similar symptoms between these conditions, corroborating findings from Medvedev and colleagues'.^10^ Bell's Palsy manifests as sudden onset of single‐sided weakness of all peripheral facial nerve branches. However, symptoms typically resolve over time in about 70% of patients.^10^ Trigeminal neuralgia induces intermittent, triggerable facial pain, but PNS causes persistent, unilateral facial pain and loss of sensation.^10^

Patients were often referred several times to multiple specialists and underwent numerous investigations before obtaining the correct diagnosis. This aligns with Zhang and colleagues' findings in which they reported that patients with PNS saw at least three different HCPs before reaching a head and neck surgeon.^16^ Participants in the current study also highlighted that certain primary HCPs like GPs with diverse cultural backgrounds might have lacked awareness about skin cancer. Limited access to health services, long‐waiting times for appointments and long‐travel distances in regional areas served as additional barriers for early diagnosis and treatments.^21^, ^33^ Despite these challenges, we also found that patients leveraged their relationships or social networks to expedite the referrals and appointments, thereby improving access to healthcare services.^21^

Given these findings, patients' gut feeling serves as a key facilitator in early diagnosis by increasing awareness and proactive help‐seeking. Future research may explore how HCPs' attitudes toward patients' awareness impact diagnostic decision‐making. Stolper and colleagues for instance found that primary HCPs can readily recognise patients' gut feelings and incorporate them into diagnostic considerations.^32^ Thus, further exploration into patients' gut feeling influencing HCP's attitudes is warranted. Exploring the barriers from the HCP's perspective is crucial, as participants noted that GPs often lacked knowledge of PNS and HCPs' struggled in obtaining their medical history. However, these findings were based on patients' subjective views. In 2012, Australia implemented the electronic health record to bridge the gap between patients and HCPs by the sharing of medical information.^34^, ^35^ Utilising electronic health records could enhance HCPs comprehensive understanding of various diseases, potentially addressing the barriers identified in this study.^35^ Hence, it is important to explore HCP viewpoints and investigate whether the electronic health records are widely used and barriers for impeding timely collect patient medical records.

### 4.1 Clinical implication

The facilitators and barriers identified by patients in this study may be addressed by HCPs and policymakers in the following ways to improve the early diagnosis and treatments of cSCC patients with PNS. Notably, there also appears to be a lack of consensus regarding the terminology of PNS and PNI, reflecting a notable divergence of opinions within scientific and medical communities.^15^, ^36^ Thus, implementing clinical practice guidelines outlining typical symptoms of PNS, and delivering education to heighten awareness among HCPs could prevent misdiagnosis. This may establish a clear pathway to timely diagnose and treat PNS.

Some previous studies have emphasised the effectiveness of clinical practice guidelines in assisting HCPs to manage diseases with high survival rates.^37^, ^38^ The Australian Government commissioned and funded Cancer Council Australia to review and update the clinical practice guidelines for keratinocyte cancer, approved by the Chief Executive Officer of the National Health and Medical Research Council in 2019.^36^ These guidelines provide comprehensive information on keratinocyte cancer, covering risk factors, prevention, appraisal, diagnosis, biomedical features, treatment, and prognosis. These guidelines explicitly address the clinical features of cSCC, indicating that a minority of cSCC cases develop PNS.^36^ The prognosis section of the guidelines provides specific information on PNS, including symptoms.^36^ These guidelines can also be used to inform cSCC patients, to increase their awareness of PNS, as patients' self‐awareness and appraisal are key facilitators in this study. Given the rarity of PNS and the challenges in diagnosis, patients were usually referred several times before an accurate diagnosis. Thus, increasing HCP's awareness via clinical practice guidelines and training programs can improve efficiency of referral systems and improve early diagnosis.

However, in regional areas, limited healthcare access poses a challenge. The COVID‐19 pandemic accelerated the implementation of telehealth for skin cancers utilising real‐time videoconferencing, with one study concluding that telehealth can assist with diagnosing skin cancers,^39^ however it is reliant on primary HCPs recognising patients' symptoms and referring them to appropriate specialists.^21^ In addition, the diagnosis of PNS requires a complete cranial nerve examination making it challenging to conduct via telehealth, which it is not yet validated. Thus, the implementation of clinical guidelines and training HCPs may promote the successful implementation of telehealth, bridging the healthcare access gaps between major cities and regional areas.

## 5 LIMITATIONS

A key limitation in this study is the potential for recall bias, as participants recounted their past experiences during interviews conducted several months or years after their treatment and disease onset. Despite the interviewers' efforts to mitigate this bias by cross‐checking participants' recollections with their medical records, recall bias may still have remained which could impact the validity of the findings. Another potential limitation is the possibility of selection bias, as participants with positive experience or favourable health outcomes regarding healthcare services could be overrepresented, while those with negative experiences could be underrepresented. However, recruitment of participants was purposive and sought to include individuals from both metropolitan and regional areas of NSW, with representation of genders and time since PNS diagnosis. This study predominantly explores patients' perspectives, and all findings are derived from their subjective viewpoints. Therefore, it is imperative to conduct further research with a focus on the HCP's perspective to provide a more comprehensive understanding of facilitators and barriers for early diagnosis and treatment of cSCC patients with PNS. Nevertheless, the interview protocol guided by the Model of Pathways to Treatment provided a comprehensive exploration of patients' opinions.

## 6 CONCLUSION

This is the first study to qualitatively explore barriers and facilitators along the diagnostic and treatment pathway for PNS. The findings in this study contribute to understanding factors that influence help‐seeking, early diagnosis, and treatment, but also identified some barriers that impede timely diagnosis and treatment. Key facilitators are patients' awareness, promoting their active engagement for help‐seeking, as they sense something unusual or have ‘gut feeling’ based on their past experiences of skin cancers. Conversely, the key barriers include challenges related to healthcare service accessibility and a limited awareness among HCPs about PNS. As patients living in regional areas experienced long‐travel time, limited access, and long‐waiting periods for appointments, resulting in geographical differences between major urban centres and regional areas, causing the delayed diagnosis and treatment. Implementing interventions such as clinical practice guidelines and training programs for HCPs can significantly increase their awareness. Additionally, telehealth could also effectively address healthcare service accessibility for residents in remote regions, however its effectiveness in this clinical circumstance needs to be established first.

## AUTHOR CONTRIBUTIONS

**Poorva Pradhan:** Conceptualization (supporting); data curation (lead); formal analysis (lead); investigation (lead); methodology (lead); project administration (lead); supervision (supporting); visualization (equal); writing – review and editing (lead). **Ke (Zoe) Wan:** Data curation (lead); formal analysis (lead); investigation (lead); methodology (supporting); project administration (supporting); visualization (equal); writing – original draft (lead). **Li Chan:** Conceptualization (equal); writing – review and editing (equal). **Tsu‐Hui (Hubert) Low:** Conceptualization (equal); methodology (equal); resources (equal); writing – review and editing (equal). **Raymond Wu:** Conceptualization (equal); resources (equal); writing – review and editing (equal). **Jenny H. Lee:** Writing – review and editing (equal). **Carsten E. Palme:** Conceptualization (equal); funding acquisition (equal); resources (equal); writing – review and editing (equal). **Jonathan R. Clark:** Conceptualization (equal); funding acquisition (equal); methodology (equal); resources (equal); writing – review and editing (equal). **Rebecca L. Venchiarutti:** Data curation (equal); formal analysis (equal); investigation (equal); methodology (lead); project administration (equal); supervision (lead); writing – review and editing (lead).

## FUNDING INFORMATION

Cancer Institute NSW (CINSW TPG 2020/2081).

## CONFLICT OF INTEREST STATEMENT

The authors declare that they have no conflicts of interest.

## ETHICS STATEMENT

Ethical and governance approval was granted by the Sydney Local Health District Human Research Ethics Committee (Protocol No X23‐0069 & 2023/ETH00364).

## Data Availability

The data that support this manuscript are available from the researchers upon reasonable request.
